# Heterocyclic Antidepressants with Antimicrobial and Fungicide Activity

**DOI:** 10.3390/molecules30051102

**Published:** 2025-02-27

**Authors:** Darya Zolotareva, Alexey Zazybin, Yelizaveta Belyankova, Sarah Bayazit, Anuar Dauletbakov, Tulegen Seilkhanov, Ulan Kemelbekov, Murat Aydemir

**Affiliations:** 1 School of Chemical Engineering, Kazakh-British Technical University, 59 Tole bi Str., Almaty 050000, Kazakhstan; zolotareva.2909@mail.ru (D.Z.); belyankovae@gmail.com (Y.B.); bayazitsarah@gmail.com (S.B.); dayletbakovanuar@gmail.com (A.D.); 2 Laboratory of Engineering Profile NMR Spectroscopy, Sh. Ualikhanov Kokshetau University, 76 Abay Str., Kokshetau 020000, Kazakhstan; tseilkhanov@mail.ru; 3 South Kazakhstan Medical Academy, 1 Al-Farabi Square, Shymkent 160019, Kazakhstan; u.kemelbekov@skma.kz; 4 Faculty of Science, Department of Chemistry, Dicle University, Diyarbakır 21280, Turkey; aydemir@dicle.edu.tr

**Keywords:** heterocyclic antidepressants, antimicrobial activity, fungicide activity, gut–brain axis, antibiotic, microbiome

## Abstract

In this review, the presence of antimicrobial and fungicidal activity in heterocyclic antidepressants was investigated. The already proven connection between the intestinal microbiome and mental health prompted the idea of whether these drugs disrupt the normal intestinal microflora. In addition, there is a serious problem of increasing resistance of microorganisms to antibiotics. In this article, we found that almost all of the antidepressants considered (except moclobemide, haloperidol, and doxepin) have antimicrobial activity and can suppress the growth of not only pathogenic microorganisms but also the growth of bacteria that directly affect mental health (such as *Lactobacillus*, *Lactococcus*, *Streptococcus*, *Enterococcus*, and *Bifidobacterium*).

## 1. Introduction

The gut and brain maintain a two-way communication system, and changes in the microbiome can affect mental health. Research has found that different compositions of gut bacteria result in different levels of psychological stress resistance. For example, the study [1] found that resilient people have microbial profiles with less inflammation and better intestinal barrier function, whereas people with higher stress levels often suffer from gut microbiome dysbiosis (an imbalance of gut bacteria that is associated with mental illnesses such as anxiety and depression). The gut microbiota generates several metabolites, including bile acids, short-chain fatty acids, and neurotransmitters such as glutamate, GABA, serotonin, and dopamine [2]. Microbiome dysbiosis may lead to decreased neurotransmitter production, which affects cognitive function and brain activity [3,4]. Bacterially produced metabolites, including short-chain fatty acids, neurotransmitters, and their precursors, affect brain concentrations of related metabolites via blood circulation, hence regulating cognitive functions [5,6]. Gut bacteria can modulate the local neurological system (e.g., the vagus nerve, enteric nerve) to speed up signals’ transmission to the brain [7]. Microbial metabolites have the ability to activate this nerve, thereby affecting cerebral functions that are associated with the regulation of emotions and stress [3,8]. There is a strong correlation between the intestinal microbiota and mental health, underscoring the importance of maintaining a healthy microbiome for optimal mental health.

In vivo studies provide substantial evidence that dysbiosis may contribute to the development of depression. The transplantation of fecal microbiota from depressed humans to healthy rodents led to the manifestation of depressive behaviors, suggesting that microbial dysbiosis may precede and contribute to the genesis of depression [9,10]. In [11], the authors compared the gut microbiomes of high- and low-anxiety groups of mice. In the high anxiety group, genera *Ruminiclostridium*, *Ruminococcaceae*, *Oscillibacter*, *Clostridiaceae*, and *Clostridiales* increased bacterial counts were noted, while the amount of the genus *Bacteroides* was reduced. Prolonged administration of *Lactobacillus rhamnosus* diminished anxiety-related behaviors and modified GABAB1b expression in the murine brain [12]. Individuals diagnosed with depression exhibit a significant increase in the species Bacteroides, accompanied by a reduction in the genera *Blautia*, *Faecalibacterium*, and *Coprococcus* [13]. Tryptophan synthetase gene expression is elevated by several gut bacteria, including *Lactobacillus*, *Lactococcus*, *Streptococcus*, and *Klebsiella*, which results in increased serotonin synthesis [14]. *Lactobacillus plantarum* [15] and *Bacillus subtilis* [16] have the ability to influence acetylcholine metabolism and generate acetylcholine precursors. In mice with infectious colitis, *Bifidobacterium longum* restored the expression of hippocampal brain-derived neurotrophic factor and anxiety-like behavior [17]. Moreover, *Bifidobacterium* and *Lactobacillus* have been documented to synthesize GABA [18], hence augmenting the inhibitory pathways inside cerebral networks. A study [19] demonstrated a notable rise in the prevalence of the species *Alistipes* in mice exposed to extended stressors. The in vivo investigation [20] demonstrated that infection with *Citrobacter rodentium* elicited anxiety-like behavior. The transplantation of fecal microbiota, mostly consisting of total coliforms, fecal coliforms, *Escherichia coli*, fecal streptococci, and enterococci, across mice exhibiting varying anxiety levels demonstrated that microbiota can modify brain chemistry and influence mammalian behavior [21]. The diagnosis of the fecal microbiome of patients with generalized anxiety disorder showed that 8 taxa demonstrated a reduced prevalence relative to the healthy controls: *Faecalibacterium*, *Eubacterium*, *Lachnospira*, *Butyricicoccus*, *Sutterella*, *Bacteroidetes*, *Ruminococcus gnavus*, and *Fusobacterium* [22]. It has been proven that *Bacteroides uniformis*, *Roseburia inulinivorans*, *Eubacterium rectale*, and *Faecalibacterium prausnitzii* positively influenced mental health maintenance through the production of short-chain fatty acids and the regulation of amino acid, taurine, and cortisol metabolic pathways [23]. The in vivo experiment [24] revealed for the first time that chronic infection with *Staphylococcus aureus* exerts a positive effect on experimental autoimmune encephalomyelitis, suggesting a dual role of infection in the etiology of multiple sclerosis. The production of extracellular adherence protein by *Staphylococcus aureus* significantly contributes to the prevention of autoimmune inflammation of the central nervous system.

Antibiotics significantly diminish the variety of gut microbiota. This reduction may result in the proliferation of specific bacteria while others are eradicated, disturbing the intricate equilibrium of the microbiome. Research indicates that a regimen of broad-spectrum antibiotics can result in a reduction of species richness, potentially requiring months or even years for complete recovery [25,26]. Some individuals may continue to experience persistent alterations in their gut microbiome composition for an extended period following antibiotic treatment. Research suggests that specific individuals may possess microbiomes that are similar to those of patients in intensive care units, which implies a transition to a less healthful microbiome state [6]. Antibiotic therapy diminishes the total diversity of gut microbiota species, resulting in the loss of certain essential taxa, which leads to metabolic alterations, heightened gut vulnerability to colonization, and the promotion of bacterial antibiotic resistance development [26].

Application of antibiotics is also a primary driver of antibiotic resistance. According to projections, the global economy could incur a cost of approximately $100 trillion by 2050 due to the potential loss of up to 10 million lives annually due to antibiotic-resistant infections [27,28]. The efficacy of current antibiotics decreases as resistance increases. It is believed that over 70% of all pathogenic bacteria are resistant to at least one antibiotic that is available for purchase [27,29]. Some microorganisms have been linked to a significant number of fatalities that are attributed to antibiotic resistance, such as *Escherichia coli*, *Staphylococcus aureus*, *Klebsiella pneumoniae*, *Streptococcus pneumoniae*, *Acinetobacter baumannii*, and *Pseudomonas aeruginosa* [30].

The composition, diversity, and functions of the intestinal microbiota can be altered by numerous currently used antidepressants [31,32]. When used together, they may improve the efficacy of conventional antibiotics by reducing their minimal inhibitory concentrations, suggesting a synergistic effect [32]. Taking into account individual microbiome profiles, additional research into the antimicrobial effects of antidepressants on the gut microbiota might be a fresh approach to improving the tailored treatment of depressive patients [32]. These results raise concerns about the development of antibiotic resistance, even though they suggest potential therapeutic advantages of antidepressants beyond their primary use. The study [33] suggests that certain antidepressants (including sertraline, duloxetine, bupropion, escitalopram, and agomelatine) can exacerbate multidrug resistance, which can complicate the treatment of infections. All tested antidepressants promoted the development of antibiotic resistance in *Escherichia coli*. The study demonstrated that the higher the dose of antidepressants, the faster the bacteria developed resistance [33]. The mechanisms by which antidepressants induce resistance include increased production of reactive oxygen species and the activation of efflux pumps that help bacteria expel antibiotics. Additionally, these drugs may stimulate genetic exchanges among bacteria, further spreading resistance [33]. Even a solo prescription of antidepressants can, due to their effect on the microflora, in the first stages of treatment have a positive impact on the patient’s mental state, but at the same time, it will cause an imbalance in his intestinal microflora, which will have a long-term negative effect on the treatment process.

In this review article, we have attempted to examine the data on the antibacterial and fungicide activity of heterocyclic antidepressants. Our goal is to find out whether antidepressants really have antimicrobial properties and therefore contribute to antibiotic resistance and mental health.

## 2. Heterocyclic Antidepressants with Antimicrobial and Fungicide Activity

### 2.1. Morpholine Moiety

**Figure 1:**
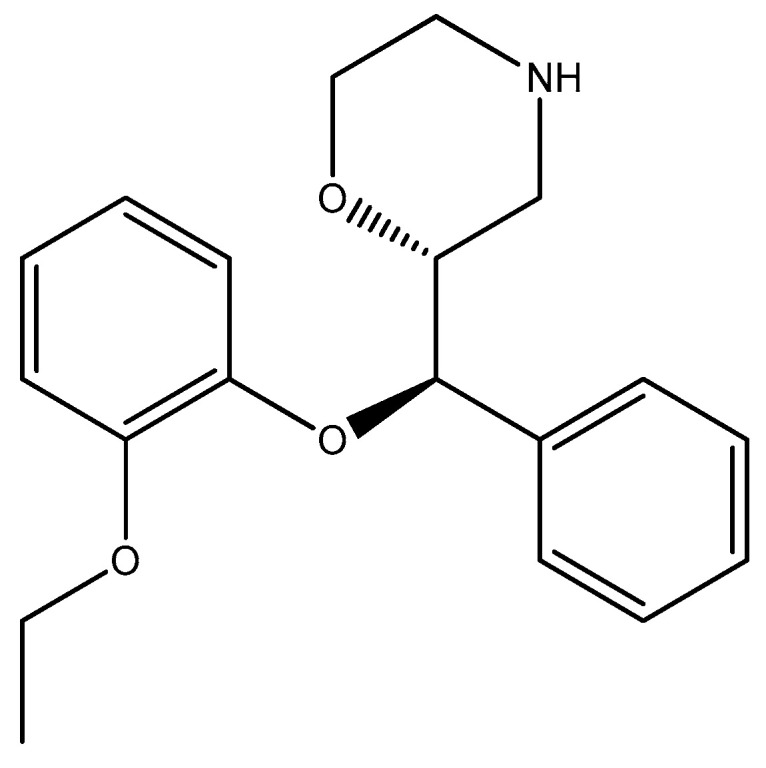
The structure of reboxetine.

Reboxetine (Figure 1) is a selective norepinephrine reuptake inhibitor (sNRI) that is sold as an antidepressant to treat major depression disorder. However, it has also been used for panic disorder and attention deficit hyperactivity disorder (ADHD) [34]. The results [35] showed that reboxetine had different levels of antibacterial activity against a variety of bacteria, including *Pseudomonas aeruginosa*, *Escherichia coli*, *Klebsiella pneumoniae*, *Proteus vulgaris*, *Yersinia enterocolitica*, *Acinetobacter baumannii*, *Staphylococcus aureus*, *Bacillus subtilis*, *Staphylococcus epidermidis*, methicillin-resistant *Staphylococcus aureus* (MRSA), vancomycin-resistant *Enterococci* (VRE), *Enterococcus faecalis*, and *Mycobacterium tuberculosis*, as well as fungi such as *Candida albicans* and *Aspergillus niger*. It was shown to produce significant reductions in metabolic activity of biofilms formed by *Candida albicans* and *Aspergillus niger* [35,36]. While it was not the most potent among the tested drugs, its ability to influence microbial resistance patterns is noteworthy [35]. In vitro studies clearly demonstrate antifungal effects against *Aspergillus fumigatus* and *Aspergillus flavus* [37].

**Figure 2:**
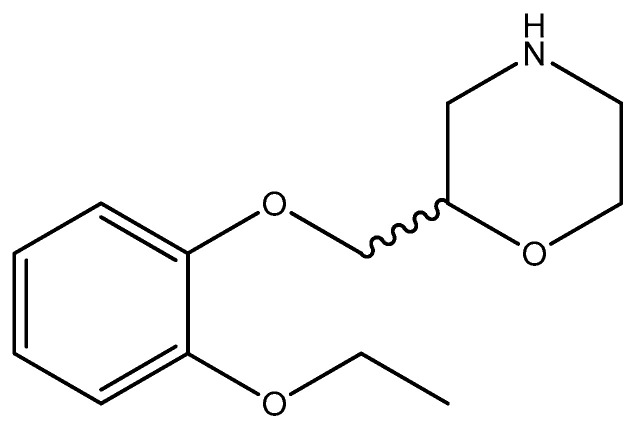
The structure of viloxazine (racemic compound; however, the (*S*)-(–)-isomer is more active).

Viloxazine (Figure 2), a selective norepinephrine reuptake inhibitor (NRI), is used in the treatment of attention deficit hyperactivity disorder (ADHD) [38]. Specifically, research has shown that certain compounds related to viloxazine were assayed for antimicrobial activity against strains such as *Escherichia coli* [39].

**Figure 3:**
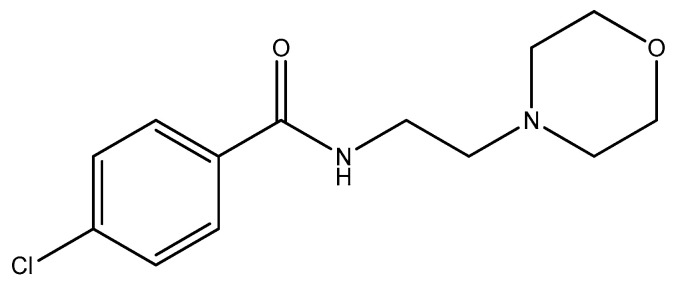
The structure of moclobemide.

Moclobemide (Figure 3) is a reversible inhibitor of monoamine oxidase A (RIMA) utilized for the treatment of depression and social anxiety [40]. Moclobemide was discovered to be entirely inert against all bacteria tested (*Pseudomonas aeruginosa*, *Escherichia coli*, *Klebsiella pneumoniae*, *Proteus vulgaris*, *Yersinia enterocolitica*, *Acinetobacter baumannii*, *Staphylococcus aureus*, *Bacillus subtilis*, *Staphylococcus epidermidis*, methicillin-resistant *Staphylococcus aureus* (MRSA), vancomycin-resistant *Enterococci* (VRE), *Enterococcus faecalis*, *Mycobacterium tuberculosis*, *Candida albicans*, *Aspergillus niger*) [35].

### 2.2. Thiomorpholine Moiety

**Figure 4:**
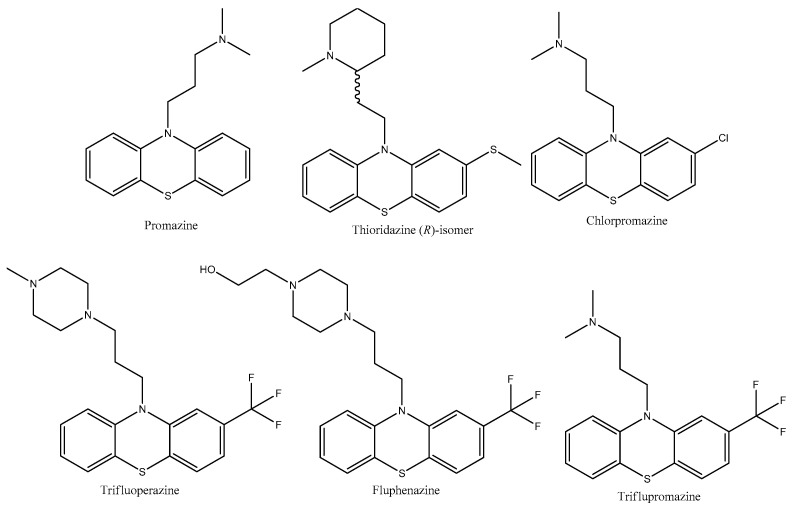
The structures of antidepressants with thiomorpholine moiety.

Promazine, thioridazine, chlorpromazine, trifluoperazine, triflupromazine, and fluphenazine (Figure 4) are antipsychotic drugs belonging to the phenothiazine group and used as tranquilizers in veterinary [41], schizophrenia, psychosis, and bipolar disorder [42,43,44,45,46].

**Figure 5:**
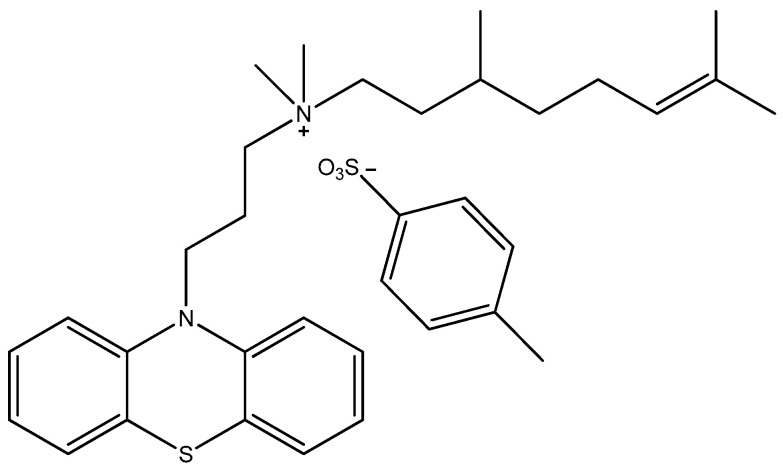
The structure of the promazine derivative JBC 1847.

Promazine (Figure 4) shows an antimicrobial effect against Staphylococcus aureus, methicillin-resistant *Staphylococcus aureus*, *Pseudomonas aeruginosa*, *Escherichia coli*, extended-spectrum beta-lactamase, *Acinetobacter baumannii*, and *Klebsiella pneumoniae* [47]. The ionic derivative of promazine (Figure 5) showed a significant enhancement in antibacterial activity. In vitro investigations demonstrated effective microbiological activity specifically against gram-positive bacteria, and it also showed strong performance in an MRSA in vivo. Furthermore, a methicillin-resistant Staphylococcus aureus strain exhibited a notably reduced level of resistance to the derivative in comparison to fusidic acid [48].

The efficacy of thioridazine (Figure 4) has been demonstrated through in vivo trials for cutaneous infections and sepsis induced by Staphylococcus aureus. The medication exhibits an antimicrobial activity against *Plasmodium falciparum* and *Trypanosoma* spp. [49]. Thioridazine has been shown in vivo to have weak activity against replicating and non-replicating bacilli (*Mycobacterium tuberculosis* H37Rv), including those with resistance to first-line drugs [35]. It helps in controlling bacillary growth in combination therapies. However, its standalone bactericidal efficacy is limited compared to more potent agents such as isoniazid [50]. Thioridazine in combination with dicloxacillin demonstrates (in vitro) significant antibacterial activity against both methicillin-sensitive and methicillin-resistant strains (MSSA and MRSA) [51].

Chlorpromazine (Figure 4) has been shown to be effective against *Staphylococcus aureus*, and it exhibits intracellular killing activity, which is particularly relevant for the treatment of infections that typically require more toxic antibiotics [52]. Recent studies [53,54] have looked into the use of chlorpromazine that has been treated with laser irradiation. In recent years, the application of laser irradiation to non-antibiotics, including phenothiazine derivatives, as well as antibiotics, has resulted in the photodegradation of the original compounds into photoproducts that may exhibit antimicrobial properties [55]. Irradiated chlorpromazine consists of a combination of chlorpromazine and its photoproducts formed in the solution following laser exposure. These include chlorpromazine sulfoxide, promazine, promazine sulfoxide, 2-hydroxy promazine, 2-hydroxy promazine sulfoxide, etc. [55]. This method has demonstrated improved antibacterial effects against *Staphylococcus aureus* ATCC 6538, methicillin-susceptible *Staphylococcus aureus*, methicillin-resistant *Staphylococcus aureus*, *Enterococcus faecium* 17-VAR, *Enterococcus faecalis* 2921, and *Bacillus subtilis* 6633 biofilms, which are a major challenge in the treatment of chronic infections. This technique improves the effectiveness of the medication against microorganisms that are usually resistant to conventional therapies [53,54]. The lower pH observed in chlorpromazine subjected to laser exposure, in contrast to the non-irradiated one, can be attributed to the photoionization process that takes place during the UV laser treatment of the solutions [53].

Trifluoperazine (Figure 4) has been demonstrated to possess potent antibacterial properties against both gram-positive and gram-negative bacteria. In the study [56], it was discovered that it inhibited 46 out of 55 strains of *Staphylococcus aureus* at doses between 10 and 50 µg/mL. It also successfully inhibited *Shigella* spp., *Vibrio cholerae*, and *Vibrio parahaemolyticus* at doses ranging from 10 to 100 µg/mL. Trifluoperazine also demonstrated moderate sensitivity against *Pseudomonas* spp. In vivo tests [56] showed that trifluoperazine offered Swiss albino mice considerable protection against lethal dosages of *Salmonella typhimurium*, which supports its potential as an antibacterial agent.

Triflupromazine (Figure 4) has shown activity against 279 strains of bacteria, with notable effectiveness against *Staphylococcus aureus*, *Escherichia coli*, and *Klebsiella pneumoniae* [57]. Triflupromazine was shown to have strong protective effects against lethal dosages of *Salmonella typhimurium* when delivered at a concentration of 30 µg/mouse in animal models, specifically Swiss albino mice. This indicates that triflupromazine has the potential to be used as a therapeutic drug beyond its antipsychotic qualities [57]. In addition, the treated mice’s organ homogenates and blood showed a decrease in the number of live bacteria, which supports its effectiveness as a microbicide [57].

Fluphenazine (Figure 4) has shown a significant antibacterial effect in a study [58] involving 482 bacterial strains. It was effective with a minimum inhibitory concentration established through nutrient agar tests. Fluphenazine at concentrations of 40–80 µg/mL inhibited 5 out of 6 *Bacillus* spp. strains and killed 95 out of 164 staphylococci. Of all the gram-negative organisms examined, *Vibrio cholerae* was the most sensitive, with 138 out of 153 strains. Fluphenazine could also inhibit 13 of 18 strains of *Vibrio parahaemolyticus* [58]. The compound has shown protective effects in animal models. For instance, when administered at doses of 1.5 and 3 µg/g of body weight in mice, fluphenazine provided substantial protection against lethal doses of *Salmonella typhimurium*, indicating its potential as an antimicrobial agent [58].

Phenothiazines have broad antimicrobial activity that is expressed against intracellular antibiotic-resistant bacteria such as *Mycobacterium tuberculosis*, *Staphylococcus aureus*, and antibiotic-resistant protozoa such as *Plasmodium falciparum*, at concentrations that are clinically relevant [59].

### 2.3. Piperidine Moiety

**Figure 6:**
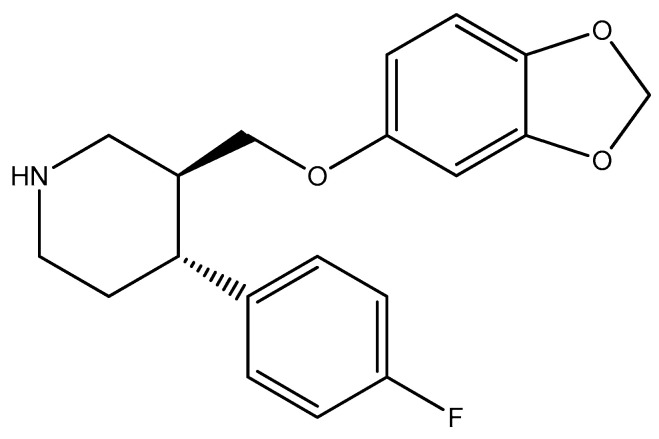
The structure of paroxetine.

Paroxetine (Figure 6) is a selective serotonin reuptake inhibitor (SSRI). Major depressive disorder, obsessive-compulsive disorder, panic disorder, social anxiety disorder, posttraumatic stress disorder, generalized anxiety disorder, and premenstrual dysphoric disorder are among the conditions it is used to treat [60]. It was proved that paroxetine has an antifungal effect against *Candida albicans*, *Candida tropicalis*, *Candida parapsilosis*, and *Candida glabrata* strains; it promoted changes in plasma and mitochondrial membrane integrity, which led to cell death through apoptosis [61,62]. Paroxetine in combination with fluconazole could have a synergistic effect against *Candida* spp. [63]. Also it presented antibacterial activity against all the ATCC standard strains (*Escherichia coli* ATCC 35218, *Escherichia coli* ATCC 25922, *Klebsiella pneumoniae* ATCC 700603, *Pseudomonas aeruginosa* ATCC 27853, *Staphylococcus aureus* ATCC 25923, *Staphylococcus aureus* ATCC 29213, Enterococcus faecalis ATCC 29212, *Enterococcus faecalis* ATCC 51299, *Staphylococcus epidermidis* ATCC 12228, *Micrococcus luteus* ATCC 7468 and *Bacillus cereus* ATCC 14579) and clinical isolates (*Staphylococcus aureus* (A), *Staphylococcus aureus* (B), *Staphylococcus aureus* (C), *Staphylococcus aureus* MDR (D) and *Acinetobacter baumanni* MDR) tested, as well as showing bactericidal activity against most tested microorganisms. However, when associated with the antibiotic ciprofloxacin, it showed a synergistic effect against two standard strains: *Staphylococcus aureus* ATCC 25923 (FICI = 0.078) and *Staphylococcus epidermidis* ATCC 12228 (FICI = 0.281), indicating potentiation of antibacterial activity [64]. Paroxetine demonstrated significant antibacterial activity against *Staphylococcus aureus* with a minimum inhibitory concentration (MIC) of 64 μg/mL. When coupled with oxacillin, it demonstrated additive interactions and bactericidal activity against *Staphylococcus aureus* strains [65]. More generally, paroxetine was evaluated against clinical strains of the ESKAPEE group, which comprises pathogens such as *Klebsiella pneumoniae* and *Escherichia coli*. It was discovered that the drug’s antibacterial action is mediated by mechanisms such as oxidative stress in bacteria and increased membrane permeability [66]. Additionally, it has been [67] discovered that paroxetine inhibits *Trypanosoma cruzi* by acting as a trypanocide.

**Figure 7:**
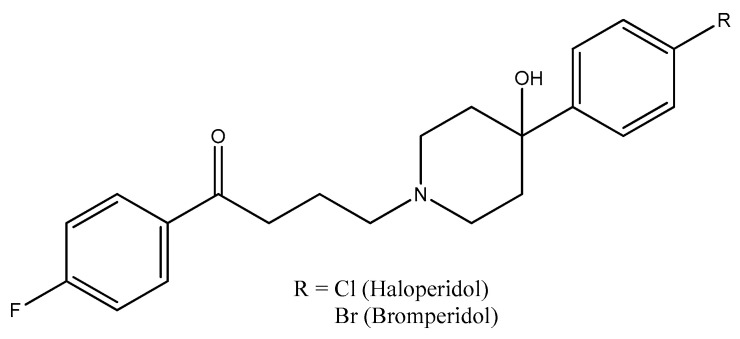
The structures of haloperidol and bromperidol.

Typical antipsychotics of the butyrophenone category, haloperidol and bromperidol (Figure 7), are used to treat schizophrenia, Tourette syndrome tics, bipolar disorder mania, delirium, agitation, acute psychosis, and alcohol withdrawal hallucinations [68,69].

Recently, it was found that haloperidol has antifungal efficacy against a strain of drug-sensitive *Candida albicans* [70]. Haloperidol does not significantly inhibit the growth of tested bacterial strains, according to study [47]. Its minimum inhibitory concentration has been found to be more than 1024 μg/mL, suggesting that it is ineffective against common infections, such as *Escherichia coli* and *Staphylococcus aureus* [47].

In contrast to haloperidol, bromperidol has demonstrated encouraging antimicrobial action against a range of pathogens, especially when it comes to therapeutic repurposing for bacterial infections. According to reports, bromperidol and spectinomycin synergistically kill mycobacteria *Mycobacterium tuberculosis* [71,72]. Bromperidol was shown to be one of several drugs that dramatically decreased Salmonella viability within macrophages in a study [73], suggesting that it works well in an intracellular setting. Furthermore, it has been shown that bromperidol can prevent *Candida* species from forming biofilms [74]. It may have an impact on bacterial resistance mechanisms, as evidenced by its capacity to increase the effectiveness of other antibiotics [71,74].

**Scheme 1:**
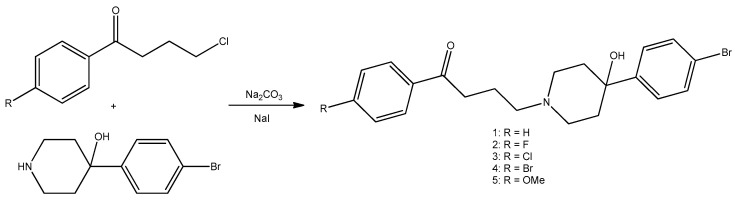
Bromperidol (**2**) and its derivatives (**1**, **3**–**5**).

Holbrook et al. [75] synthesized four bromperidol derivatives (Scheme 1) and tested their combinational antifungal activities with clinical azole antifungals. Fluconazole, itraconazole, ketoconazole, posaconazole, and voriconazole were tested against seven strains of *Candida albicans*, one non-albicans *Candida* (*Candida glabrata*), and one filamentous fungus (*Aspergillus terreus*). Posaconazole synergized more with compounds **1**–**5** than voriconazole. Cytotoxicity testing revealed voriconazole combo therapy may be better for mammalian cytotoxicity. Bromperidol derivatives in combination with clinically relevant azoles can synergistically inhibit fungal growth and reduce the amount of azoles needed to achieve an equivalent antifungal effect, reducing the toxicity and side effects of high azole concentrations.

### 2.4. Piperazine Moiety

**Figure 8:**
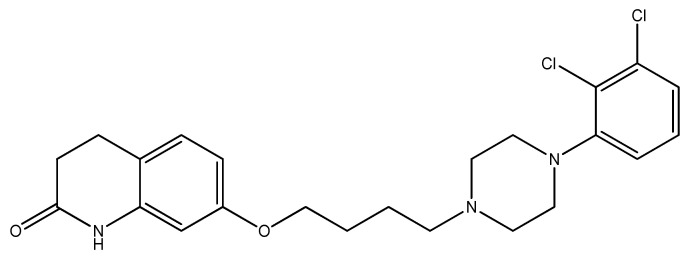
The structure of aripiprazole.

Aripiprazole (Figure 8) is an atypical antipsychotic, a partial agonist of serotonin and dopamine receptors, contains both piperazine and piperidine fragments, and is used to treat schizophrenia, bipolar disorder, and ASD irritability [76,77]. At lower concentrations, it demonstrated superior efficacy compared to ketoconazole, effectively inhibiting biofilm formation, yeast-to-hyphal transition, and flocculation. Conversely, at elevated concentrations, it caused disruption of lipid rafts and induced membrane damage of *Candida albicans* [78]. Aripiprazole also exhibits antibacterial activity against gut microbiota [16]. This study revealed a high antibacterial sensitivity of aripiprazole against *Lactobacillus reuteri* ATCC 23272, *Lactobacillus rhamnosus* ATCC 53103, *Lactobacillus casei* ATCC 393, *Bifidobacterium animalis* ATCC 25527, *Enterococcus faecium* ATCC 35667, *Eubacterium rectale* ATCC 33656, Faecalibacterium prausnitzii ATCC 27768, Bacteroides fragilis ATCC 2528, Pseudomonas aeruginosa ATCC 27853, Escherichia coli ATCC 25922, *Clostridium leptum* ATCC 29065, and *Akkermansia muciniphila* ATCC BAA-835 [32].

### 2.5. Pyridine Moiety

**Figure 9:**
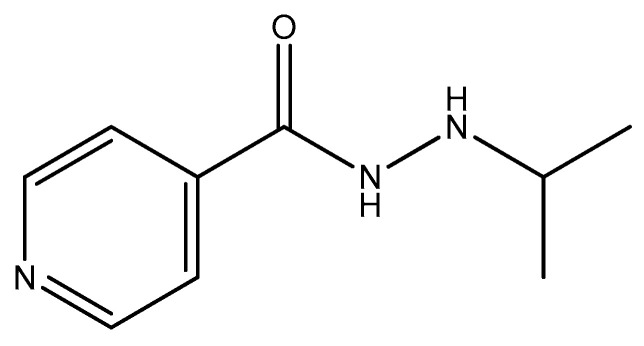
The structure of iproniazid.

Iproniazid (Figure 9) is classified as a non-selective, irreversible monoamine oxidase inhibitor (MAOI) belonging to the hydrazine class [79]. Iproniazid and its parent compound, isoniazid, were initially developed for the treatment of tuberculosis [80]. The product was removed from the market due to its potential hepatotoxic effects [81]. The clinical trials [82] indicated that in patients with advanced tuberculosis, there was an earlier manifestation of reduced fever, decreased sputum, weight gain, and regressive changes in the tuberculous process on X-ray compared to a comparable group of patients treated solely with isoniazid. Regrettably, the emergence of toxic symptoms led to the early cessation of iproniazid.

**Figure 10:**
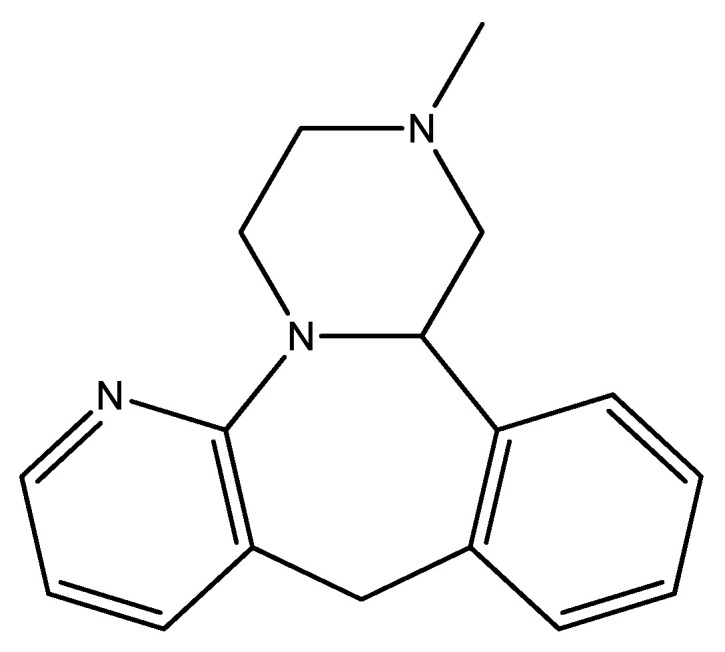
The structure of mirtazapine.

Mirtazapine (Figure 10) is an atypical tetracyclic antidepressant (TCA), noradrenergic and specific serotonergic antidepressant (NaSSA), and is used primarily to treat depression [83]. Mirtazapine has been found to have a substantial inhibitory influence on the growth of the normal gut microbiota (*Staphylococcus aureus* ATCC 25923, *Escherichia coli* ATCC 25922, *Candida albicans* ATCC 24433, *Bifidobacterium* 791, *Enterococcus faecalis* ATCC 29212, *Lactobacillus rhamnosus* ATCC 53103). Specifically, it has shown the greatest inhibitory effects against *Lactobacillus rhamnosus* and *Candida albicans* in vitro, suggesting potential implications for gut health and microbiome balance [84,85].

**Figure 11:**
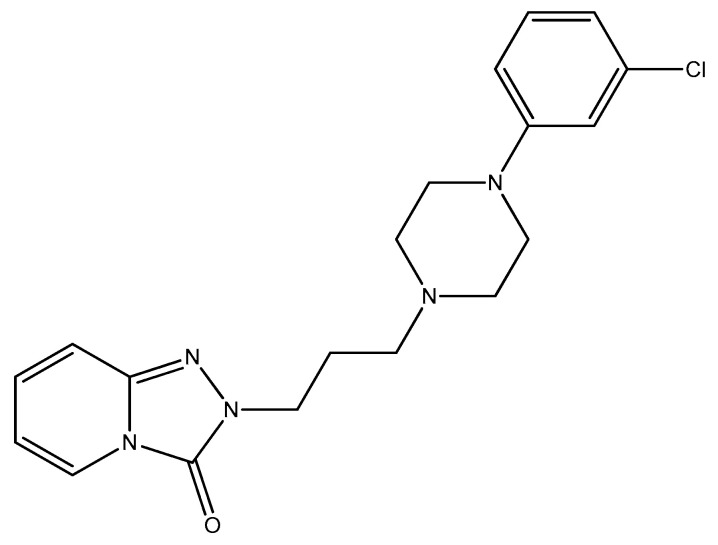
The structure of trazodone.

Trazodone (Figure 11) is an antidepressant that is prescribed to alleviate insomnia, anxiety disorders, and major depressive disorder [86]. The study [35] indicated that trazodone exhibited antibacterial activity against *Yersinia enterocolitica*, *Proteus vulgaris*, and *Acinetobacter baumannii*. Research [87] has also examined trazodone’s effects on the gut microbiome in canines. A study revealed that trazodone did not substantially affect the variety or composition of gut microbiota in dogs administered the drug relative to untreated pups. This indicates that although trazodone may possess certain antibacterial capabilities, its effect on the gut microbiota is minimal.

### 2.6. Seven-Membered Heterocycles

**Figure 12:**
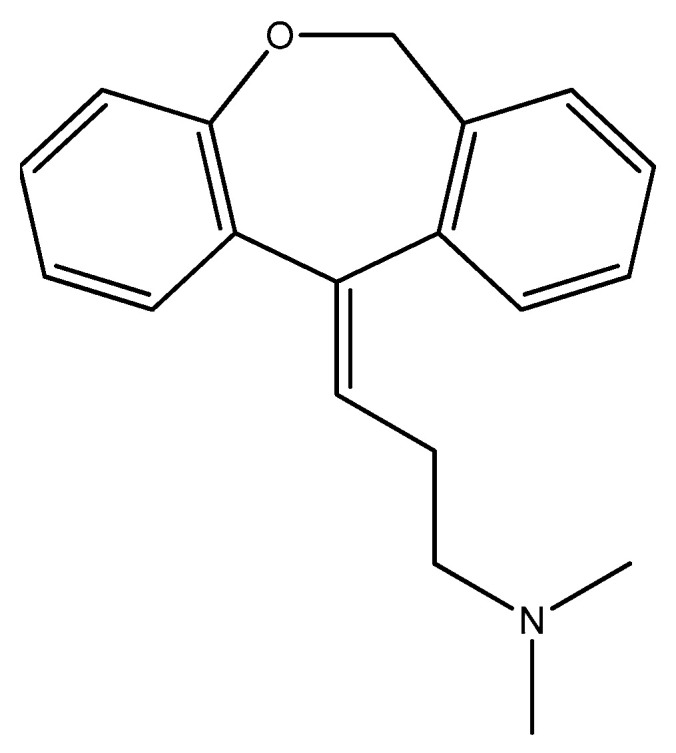
The structure of doxepin.

Doxepin (Figure 12) is a tricyclic antidepressant (TCA) utilized for the treatment of major depressive disorder, anxiety disorders, chronic hives, and insomnia [88]. Prevents the development of hyphae and biofilm, effectively eliminating cells within an established yeast biofilm (*Candida albicans*, *Candida glabrata*, *Candida parapsilosis*, *Candida krusei*, *and Candida utilis*) [89]. A study [90] demonstrated that doxepin shows no antibacterial activity against *Bacillus subtilis* and *Pseudomonas aeruginosa*.

**Figure 13:**
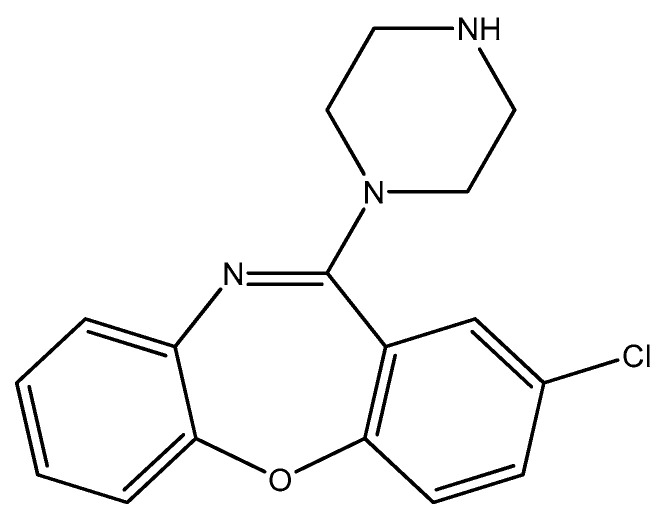
The structure of amoxapine.

Amoxapine (Figure 13) is a tricyclic antidepressant (TCA) that reduces the uptake of serotonin and noradrenaline [91]. An in vivo study [92] demonstrates increased survival of mice infected with pneumonic plague (*Yersinia pestis*). Amoxapine reduced GUS-mediated hydrolysis of β-Dglucuronide, lowering *Salmonella*’s energy source for proliferation [93]. Research [94] indicates that while amoxapine does not exhibit significant direct antibacterial effects against *Mycobacterium tuberculosis*, it inhibits intracellular mycobacterial survival by inducing autophagy. Amoxapine’s efficacy against mycobacterial infection further supports its potential as a host-directed therapy for tuberculosis treatment. Given the increased concern over drug-resistant forms of tuberculosis, this approach is particularly helpful. Modern mycobacterium tuberculosis strains need new treatments that target host factors necessary for bacteria to survive [94].

**Figure 14:**
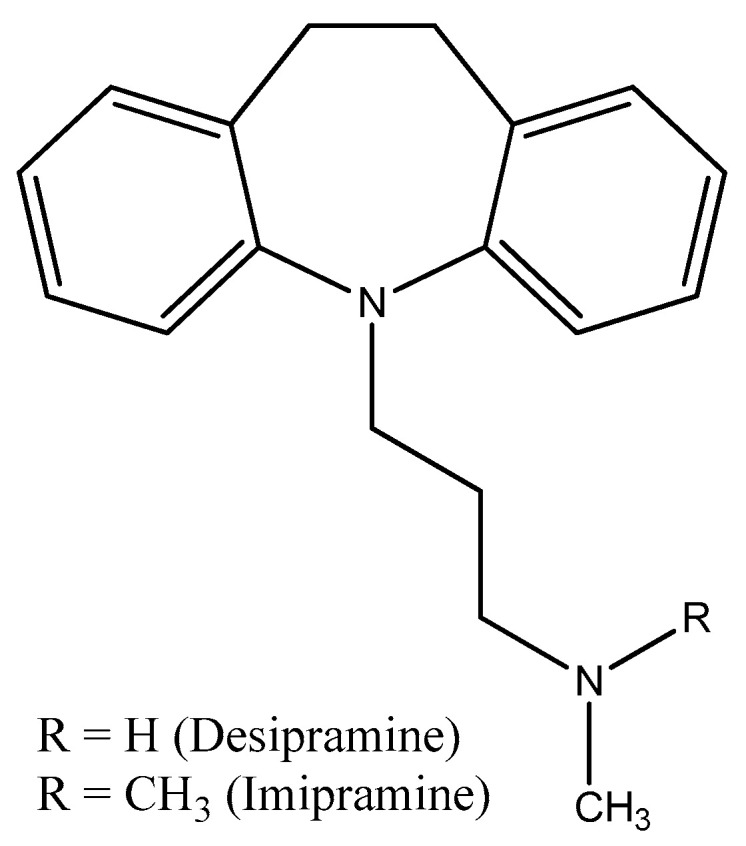
The structures of imipramine and desipramine.

Imipramine and desipramine (Figure 14) are tricyclic antidepressants (TCA) primarily utilized in the management of depression [95,96]. Imipramine demonstrates efficacy in the treatment of anxiety and panic disorder [96]. Prevents the development of hyphae and biofilm, effectively eliminating cells within an established yeast biofilm (*Candida albicans*, *Candida glabrata*, *Candida parapsilosis*, *Candida krusei*, and *Candida utilis*) [89]. Imipramine has been shown to effectively cure plasmids of *Escherichia coli* K12 at 37 °C and demonstrated an antimicrobial effect at concentrations above 200 µg/mL, killing over 90% of the bacteria within 60 min [97].

In a study [32], desipramine demonstrated considerable inhibitory effects against 12 commensal bacterial strains (*Lactobacillus reuteri* ATCC 23272, *Lactobacillus rhamnosus* ATCC 53103, Lactobacillus casei ATCC 393, *Bifidobacterium animalis* ATCC 25527, *Enterococcus faecium* ATCC 35667, Eubacterium rectale ATCC 33656, *Faecalibacterium prausnitzii* ATCC 27768, *Bacteroides fragilis* ATCC 25285, *Pseudomonas aeruginosa* ATCC 27853, Escherichia coli ATCC 25922, *Clostridium leptum* ATCC 29065, *Akkermansia muciniphila* ATCC BAA-835). This suggests that desipramine can act as both bacteriostatic and bactericidal against specific gut bacteria.

**Figure 15:**
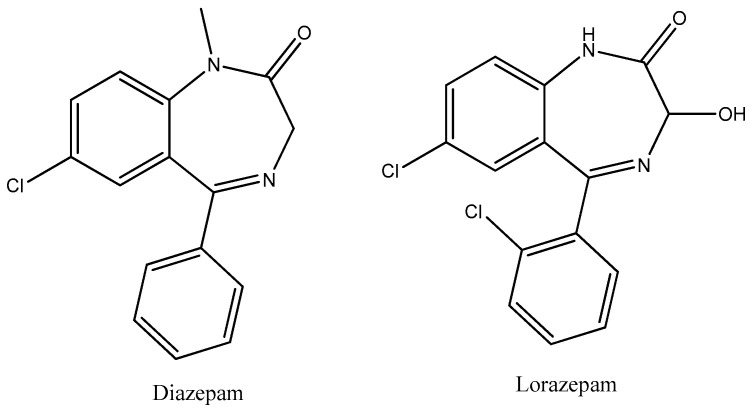
The structures of diazepam and lorazepam.

Diazepam and lorazepam (Figure 15) are anxiolytics belonging to the benzodiazepine class and are utilized in the treatment of anxiety (including anxiety disorders), sleep disturbances, severe agitation, active seizures such as status epilepticus, alcohol withdrawal, and nausea and vomiting induced by chemotherapy [98,99]. They both inhibit growth, hyphae formation, and biofilm growth of *Candida albicans* [100,101]. Study [102] demonstrates that diazepam possesses considerable antifungal efficacy against *Candida* species, particularly *Candida albicans*. This activity is especially significant against planktonic cells and biofilms produced by these fungi, causing loss of membrane integrity, mitochondrial depolarization, increased reactive oxygen species (ROS) production, DNA damage, and ultimately cell apoptosis [102]. Lorazepam has been shown to inhibit the growth, hyphae formation, and biofilm development of *Candida albicans* [74].

Diazepam inhibits the growth of Enterococcus faecium, Staphylococcus aureus, Klebsiella pneumoniae, Acinetobacter baumannii, Pseudomonas aeruginosa, and Enterobacter spp., according to a study [103]. It works synergetically with ciprofloxacin against ESKAPE pathogens, which are a type of bacteria that are known to be resistant to antibiotics.

**Figure 16:**
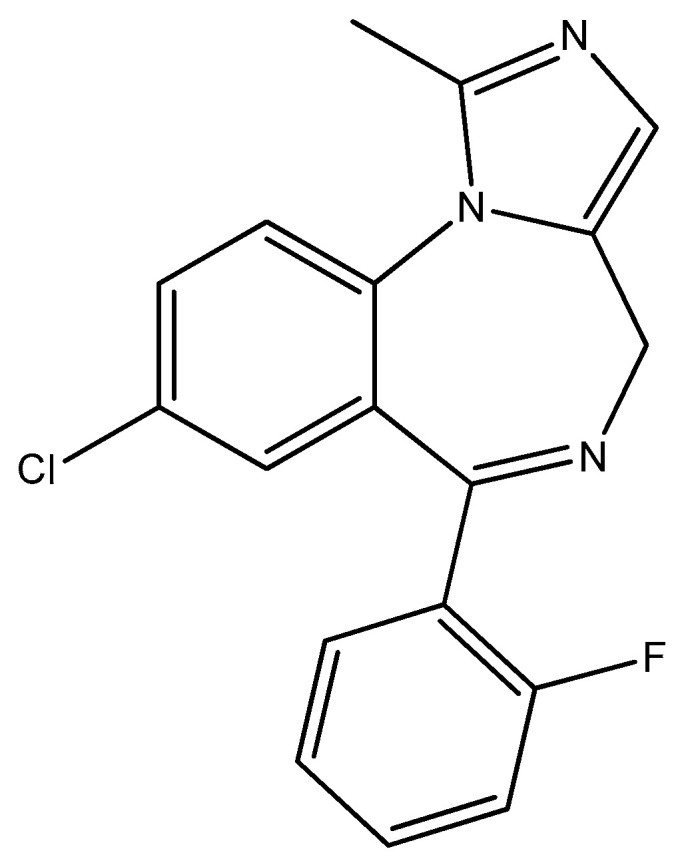
The structure of midazolam.

Midazolam (Figure 16) is a benzodiazepine utilized for anesthesia, premedication prior to surgical anesthesia, procedural sedation, and the management of acute agitation. It induces drowsiness, mitigates anxiety, and produces anterograde amnesia [104]. Inhibits the proliferation of bacteria (*Candida albicans*, *Staphylococcus aureus*, *Enterococcus faecalis*, *Escherichia coli*, *Pseudomonas aeruginosa*, *Acinetobacter baumannii*) [100,105]. *Staphylococcus aureus*, *Escherichia coli*, *Enterococcus faecalis*, *Klebsiella pneumoniae*, and *Pseudomonas aeruginosa* have all been demonstrated to exhibit inhibitory effects by midazolam [106]. The growth of *Escherichia coli* and *Pseudomonas aeruginosa* was wholly inhibited by midazolam concentrations of approximately 256 µg/mL in the study [107]. Midazolam also exhibited antifungal properties against planktonic cells and reduced the viability of *Candida* spp. biofilms [108].

**Figure 17:**
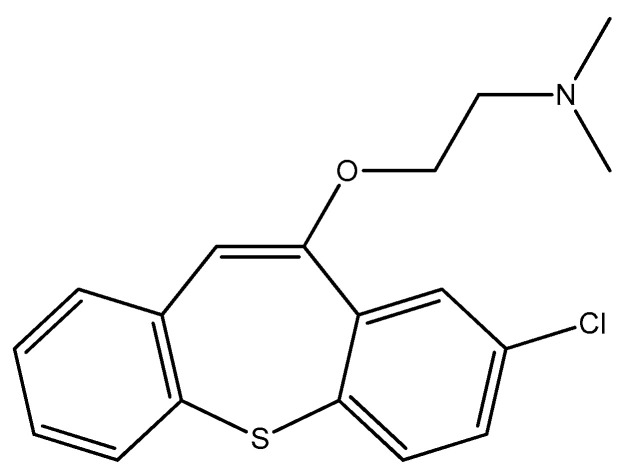
The structure of zotepine.

Zotepine (Figure 17) is an atypical antipsychotic medication intended for the treatment of acute and chronic schizophrenia [109]. Zotepine has shown effectiveness against *Candida albicans*, indicating its potential to inhibit biofilm development, which is crucial for the pathogenicity of this yeast. It has been noted to inhibit the growth of *Staphylococcus aureus* [74].

### 2.7. Five-Membered Heterocycles

**Figure 18:**
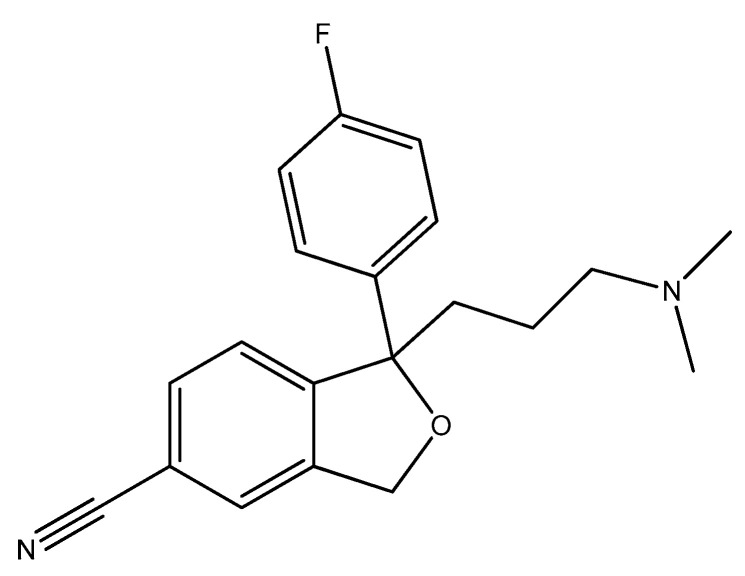
The structure of citalopram (racemic mixture).

Citalopram (Figure 18) is a selective serotonin reuptake inhibitor (SSRI) antidepressant that is used to treat major depressive disorder, obsessive-compulsive disorder, panic disorder, and social phobia. It is a racemic variant of the drug [110]. It has demonstrated antibacterial activity against various bacterial strains of *Staphylococcus aureus* ATCC 6538 and *Escherichia coli* ATCC 8739 and clinical isolates such as *Salmonella typhi*, *Klebsiella pneumoniae*, and *Enterococcus faecalis* [111]. Moreover, citalopram increased the activity of antibiotics (including levofloxacin, moxifloxacin, and gentamicin) in a concentration-dependent manner.

**Figure 19:**
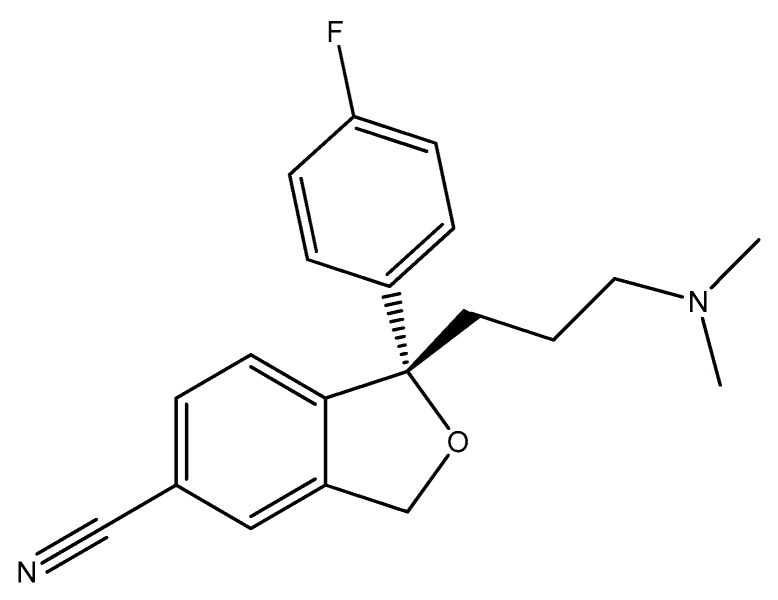
The structure of escitalopram ((*S*)-enantiomer).

Escitalopram (Figure 19) is a (*S*)-enantiomer of citalopram, an antidepressant belonging to the selective serotonin reuptake inhibitor (SSRI) class, primarily utilized for the treatment of major depressive disorder and generalized anxiety disorder [112]. Escitalopram has demonstrated significant antimicrobial effects, particularly against *Escherichia coli*, *Enterococcus faecalis*, and other strains. In the study [84], it reduced the growth rate of *Escherichia coli* by approximately 81% at a concentration of 200 μg/mL. It has been noted that escitalopram can completely inhibit the growth of certain strains, such as *Escherichia coli* and *Lactobacillus rhamnosus* at concentrations around 600 μg/mL [32].

**Figure 20:**
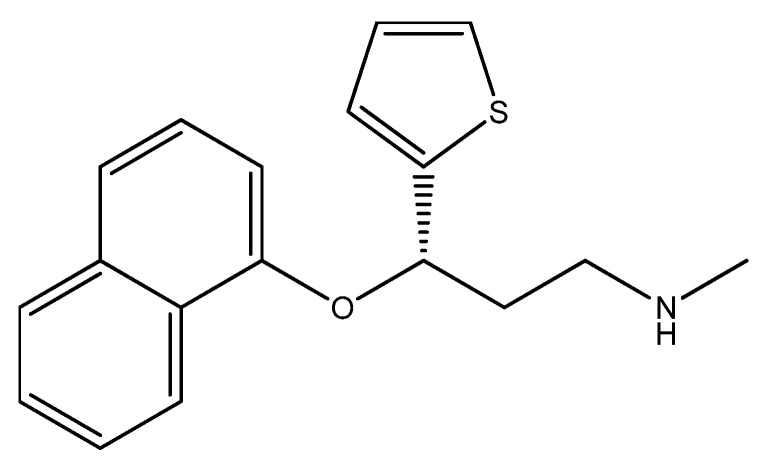
The structure of duloxetine.

Duloxetine (Figure 20) functions as a serotonin–norepinephrine reuptake inhibitor (SNRI) and is utilized in the treatment of major depressive disorder, generalized anxiety disorder, obsessive-compulsive disorder, fibromyalgia, neuropathic pain, and central sensitization [113]. At a concentration of 200 μg/mL, duloxetine reduced the growth rate of Bifidobacterium bifidum by approximately 70% and had similar effects on *Lactobacillus rhamnosus*, *Escherichia coli*, and *Enterococcus faecalis*, with reductions in growth rates ranging from 76% to 85% compared to control groups [84].

## 3. Conclusions

This review has shown that heterocyclic antidepressants may have antimicrobial and fungicidal activity. Among all the heterocyclic antidepressants considered, only moclobemide, haloperidol, and doxepin did not show antimicrobial activity (however, they all showed fungicidal activity). Aripiprazole, reboxetine, chlorpromazine, triflupromazine, fluphenazine, paroxetine, diazepam, midazolam, mirtazapine, desipramine, citalopram, escitalopram, and duloxetine inhibit the growth of the genus *Lactobacillus*, *Lactococcus*, *Streptococcus*, *Enterococcus*, and *Bifidobacterium*, which participate in the synthesis of GABA, tryptophan, serotonin, and acetylcholine and are proven to improve the course of anxiety and depression. Aripiprazole and desipramine inhibit the growth of *Bacteroides fragilis*, whose levels have been found to be significantly elevated in people with depression. At the same time, they suppress the growth of *Faecalibacterium prausnitzii*, the deficiency of which is associated with depression. Reboxetine, promazine, triflupromazine, paroxetine, diazepam, midazolam, and citalopram inhibit the growth of *Klebsiella pneumoniae*, which is known to increase the gene expression of tryptophan synthetase. Reboxetine, promazine, thioridazine, chlorpromazine, triflupromazine, trifluoperazine, fluphenazine, paroxetine, mirtazapine, diazepam, midazolam, zotepine, and citalopram inhibit the growth of the genus *Staphylococcus* (*Staphylococcus epidermidis* and *Staphylococcus aureus*), which significantly contributes to the prevention of autoimmune inflammation in the central nervous system. Reboxetine, chlorpromazine, fluphenazine, and doxepin inhibit the growth of *Bacillus subtilis*, which can produce acetylcholine precursors and affect acetylcholine metabolism.

While the dual action of antidepressants may help reduce the dosage of antibiotics when treating patients already taking antidepressants, it is of much greater concern about the increasing antibiotic resistance and the impact on mental health (due to the brain-gut connection) that is being promoted by this dual action of non-antibiotics. The findings highlight an urgent need for further research to understand how antidepressants might contribute to antibiotic resistance in real-world settings, including in vivo experiments (mice and humans) and environmental contexts.

## Data Availability

The data presented in this study are available upon request from the corresponding author or first author.
